# Big-Science facilities in Europe need greater coordination of resources

**DOI:** 10.1107/S2052252516013580

**Published:** 2016-08-31

**Authors:** Dimitri N. Argyriou

**Affiliations:** a European Spallation Source ERIC, PO Box 117, SE-221 00 Lund, Sweden

**Keywords:** Big-Science facilities, ILL, ESRF, ESS

## Abstract

The leading role in science played by crystallography is heavily dependent on Big-Science facilities. The need for Europe-wide coordination of operational resources in Big Science is discussed with particular reference to neutron sources.

Big-Science facilities such as the ILL and ESRF are important and necessary tools that ensure the leading role in science played by the crystallographic community. Indeed IUCr journals have been critical in chronicling key results, major developments and breakthroughs accomplished in crystallography that would have been impossible without the Big-Science facilities, FELs being one of the most recent examples. Unquestionably, Europe has been extremely successful in building Big-Science facilities in this collaborative fashion. But building facilities is one thing. Financing their operation is quite another.

Operations funding is by far the largest fraction of the investment in Big-Science projects that have an average lifetime of 40 years. The European Strategy Forum on Research Infrastructures (ESFRI) has played a laudable and effective role in coordinating the prioritization of Big-Science construction projects in Europe, and more recently has gone a step further by providing a strategy report for European research infrastructures (see http://www.esfri.eu/roadmap-2016). Infrastructures here may vary from particle physics to telescopes but do include facilities relevant to crystallography such as the ESS in Sweden, ESRF in France and XFEL in Germany . While these actions are clearly needed on a European scale, we still lack a forum that coordinates the operational resources needed for these facilities with the aim of establishing a path for sustainable funding that ensures continual scientific success.

The current need for Europe-wide coordination for operational resources in Big-Science is plainly obvious in the world of neutron sources. The unique properties of neutrons make them an important and irreplaceable tool in advanced materials science, providing matchless information to other techniques. Indeed crystallography thrives in neutron sources making contributions ranging from magnetic and engineering materials, to polymers and biology. It is for these contributions to science and industry that neutron facilities continue to attract substantial investments worldwide.

For decades Europe has enjoyed supremacy in the world of neutron science due to its highly integrated two-tier network. Smaller facilities have supported the larger more powerful international sources such as the ILL in France and ISIS in the UK, acting as ideas incubators and training centers, developing methods, technology and instruments. This friendly and stimulating eco-system is the key of this European success story! The challenge for Europe is that the first tier of facilities is eroding rapidly, with medium flux nuclear reactors reaching the end of their operational life. In a post Fukushima world, even the ILL has been hugely affected by new and essential regulations, and much has been spent to successfully ensure its safety and compliance.

To make matters more complicated, the BER-II reactor in Berlin, Germany, and the Orphé reactor in Saclay, France, will cease to operate at the end of this decade. These facilities serve both national and European communities and their closure represents a significant blow to European crystallography as well as a loss of hard-to-get expertise. Existing national facilities with significant capacities and capabilities, such as the Heinz Maier-Leibnitz Zentrum in Munich, Germany, do need extra resources to support the local and regional communities in the aftermath of these closures.

These decisions although fully justified from a national and institutional perspective, do have consequences far beyond their national borders and put in jeopardy the sustainability of a thriving European crystallographic community. The closure of these facilities will not be able to be absorbed easily by existing neutron centres and even more so if the ILL ceases operations at some point in the next decade. Indeed ESFRI’s neutron landscape group in its recently published report (Carlile & Petrillo, 2016 ▸), paints a dark picture for the future of neutrons; 30% of the neutron instrument availability in Europe may close by the middle to the end of the next decade and the advent of ESS will not be enough or in time to counter this dramatic decrease.

While various scenarios delay or stretch out the drop in instrument availability, the consequences for the community are serious. These losses will undoubtedly affect the size of the user community as it would be harder for students to pursue neutron experiments and research groups will turn to other techniques. This potentially can undermine the investment in ESS, as the community that will be supporting it most will be getting weaker and smaller at a critical time.

Certainly, funding bodies and ministries struggle to support the operating budget of European Big Science, often balancing investment from one facility or community against another by necessity. However, priorities are driven more here by national needs and less by strategic needs focused in ensuring the success of European science. While clearly this reflects the diversity of Europe, at the same time we should see what we could learn from the USA where a single agency plays such a prioritization and coordination role across a rich portofolio of Big-Science facilities.

There are many ideas on how to rebuild the first tier of neutron sources in Europe (Feder, 2016 ▸), using, for example, compact accelerator driven facilities, but these will take time to become fully mature as a concept and be built in sufficient numbers to serve the needs of the wider crystallographic community. There is no doubt that neutron sources are undergoing a transition from reactor-based to accelerator-based technologies and it is vitally important to manage this transition well so the user community continues to thrive. This takes forward thinking and planning and in some cases actively deciding to extend operations in reactor facilities enough for such a transition to occur in an structured manner. However, with complex decision-making processes distributed amongst different funding agencies, sensible management of such transition may be difficult at present. Irrespective of these difficulties in reaching decisions, Europe undoubtedly needs a clear and coherent strategy to support the neutron community during these transition years and to rebuild the first tier neutron sources on a new technological foundation.

While ESFRI has a wide mandate for prioritizing needs for the construction of Big-Science projects, the same wide consultative mechanism might be successful in prioritizing resources for existing facilities. If such a mandate would be established, it should go further and track the progress and scientific competitiveness of Big-Science facilities and advise on their long-term viability, by ensuring that scientific needs are met. With such a mechanism we would be equipped to manage the difficulties resulting from transitions in scientific capacity and capability such as the neutron community is currently facing. Establishment of such a mandate would be a bold and courageous step by European governments; but most importantly it would ensure that scientific communities, such as crystallography continue to thrive in Europe.
